# Epigenetic Silencing of the Circadian Clock Gene CRY1 is Associated with an Indolent Clinical Course in Chronic Lymphocytic Leukemia

**DOI:** 10.1371/journal.pone.0034347

**Published:** 2012-03-28

**Authors:** Maher Hanoun, Lewin Eisele, Masako Suzuki, John M. Greally, Andreas Hüttmann, Semra Aydin, René Scholtysik, Ludger Klein-Hitpass, Ulrich Dührsen, Jan Dürig

**Affiliations:** 1 Department of Hematology, University Hospital, Essen, Germany; 2 Institute of Cell Biology, University of Essen, Duisburg, Germany; 3 Department of Genetics, Albert Einstein College of Medicine, Bronx, New York, United States America; University of Bonn, Institut of Experimental Hematology and Transfusion Medicine, Germany

## Abstract

Disruption of circadian rhythm is believed to play a critical role in cancer development. Cryptochrome 1 (CRY1) is a core component of the mammalian circadian clock and we have previously shown its deregulated expression in a subgroup of patients with chronic lymphocytic leukemia (CLL). Using real-time RT-PCR in a cohort of 76 CLL patients and 35 normal blood donors we now demonstrate that differential CRY1 mRNA expression in high-risk (HR) CD38+/immunoglobulin variable heavy chain gene (IgVH) unmutated patients as compared to low-risk (LR) CD38−/IgVH mutated patients can be attributed to down-modulation of CRY1 in LR CLL cases. Analysis of the DNA methylation profile of the CRY1 promoter in a subgroup of 57 patients revealed that CRY1 expression in LR CLL cells is silenced by aberrant promoter CpG island hypermethylation. The methylation pattern of the CRY1 promoter proved to have high prognostic impact in CLL where aberrant promoter methylation predicted a favourable outcome. CRY1 mRNA transcript levels did not change over time in the majority of patients where sequential samples were available for analysis. We also compared the CRY1 expression in CLL with other lymphoid malignancies and observed epigenetic silencing of CRY1 in a patient with B cell acute lymphoblastic leukemia (B-ALL).

## Introduction

Accumulating epidemiological and genetic evidence indicates that disruption of circadian rhythms may increase the susceptibility for developing cancer including non-Hodgkin lymphoma (NHL) and also adversely affects tumor progression [1]–[5]. At the molecular level, several genes constituting the clock machinery have been found to establish functional interplays with regulators of the cell cycle, and disrupted expression of these genes has been shown to result in aberrant cell proliferation [2], [3], [6], [7]. In a previous study we first described disturbances in the molecular circadian machinery of CLL cells and hypothesized that these alterations may play a role in the molecular pathogenesis of the disease [8]. In particular, we found that the core circadian gene CRY1 is up-regulated in samples from high-risk ZAP-70+/CD38+ CLL patients as compared to their ZAP-70−/CD38− counterparts which are characterized by a more benign clinical course [8], [9]. Therefore, based on these data we and others have proposed that CRY1 may serve as a novel prognostic factor which could be useful for the clinical management of CLL patients [8]–[11]. Functionally, CRY1 has been shown to be essential to the maintenance of circadian rhythm because of its role in the negative arm of the circadian feedback loop [12]. However, independent of its circadian function it may have an additional role as a transcriptional regulator of a number of genes involved in cell metabolism and proliferation [4], [6], [12], [13]. In the current study we further investigated the role of CRY1 by comparing its expression pattern in molecularly defined CLL subgroups to that of B cells obtained from the peripheral blood of normal donors. Furthermore, we aimed to determine the molecular mechanism(s) underlying deregulated CRY1 expression in CLL.

## Materials and Methods

### Patients and samples

Peripheral blood samples from 76 CLL patients and 35 normal donors (ND) were analyzed after obtaining written consent according to our institutional guidelines, approved by the Ethics Commission of the University of Essen-Duisburg. The diagnosis of CLL required a persistent lymphocytosis of more than 5.0×10^9^/l and a typical CD19^+^, CD20^+^, CD5^+^, CD23^+^, Ig light chain (κ or λ light chain) restricted immunophenotype as revealed by flow cytometry of peripheral blood cells [14]. Blood samples from CLL patients and ND were drawn in the morning hours and immediately processed. Peripheral blood mononuclear cells (PBMC) were isolated by Lymphoprep density gradient centrifugation (Invitrogen, Karlsruhe; Germany) and cryopreserved until further analysis. Patient selection for this study was based on the availability of viably frozen DMSO preserved PBMC and/or freshly isolated total RNA stored in our CLL cell bank. Clinical and laboratory data of the study population are shown in Table 1.

**Table 1:** Patient characteristics.

Parameter		No. of patients (%)
Total No.		76
Sex	male	53 (70)
	female	23 (30)
Age, years	median	61
	range	32–86
Follow-up, months	median	78.0
Binet Stage at diagnosis	A	53 (70)
	B	17 (22)
	C	4 (5)
	n.a. *	2 (3)
Binet Stage at last follow-up	A	24 (32)
	B	14 (18)
	C	12 (16)
	n.a. *	26 (34)
CD38 expression	<20%	40 (52)
	≥20%	36 (48)
IgVH mutational status	unmutated	21 (28)
	mutated	21 (28)
	n.a. *	34 (44)
FISH cytogenetics	favorable	33 (43)
	unfavorable	22 (29)
Treatment history	untreated	42 (55)
	treated	32 (42)

* n.a., not available.

Five healthy donors and a subgroup of 57 CLL patients were subjected to DNA methylation analysis. Patient characteristics of this subgroup are shown separately in Table S1. For this set of experiments CD19+ cells were positively selected from PBMCs employing the EasySep Human CD19 Selection Kit (StemCell Technologies, Canada) according to the manufacturer's instruction resulting in a >90% purity of CD19+ B cells. DNA was isolated from the immunomagnetically purified CD19+ cell fraction using the DNeasy Blood and Tissue Kit (Qiagen, Hilden, Germany).

### Flow cytometry

Cell surface expression of CD38 was examined by flow cytometry using a previously described panel of fluorochrome-labeled monoclonal antibodies (CD38 PE, clone HB7) in a standard three-color flow cytometry approach using a 20% cut-off to define CD38 positivity [15], [16].

### Fluorescence in situ hybridization (FISH)

Prognostically relevant anomalies of chromosomal regions 11q, 13q, and 17p, and of chromosome 12 were assessed by fluorescence in situ hybridization, as described previously [16].

### Real-time reverse-transcriptase-PCR (qRT-PCR)

Total RNA from PBMC was extracted with RNeasy Midi Kit (Qiagen, Hilden, Germany) and spectrophotometrically quantified as previously described [8]. First strand DNA was synthesized from 1 µg of RNA using oligo(dT) primers employing a commercially available kit (RT-PCR Amplimers, Becton Dickinson, Heidelberg, Germany) according to the manufacturer's instructions. Real-Time PCR was performed with the ABI Prism 7900HT Sequence Detector (Applied Biosystems, Foster City, CA, USA) using the TaqMan Universal PCR Master Mix protocol (Applied Biosystems). Specific assays for CRY1 (Hs00172734_m1, Assays-on-Demand Gene Expression System, Applied Biosystems), BMAL1 (Hs00154147_m1, Assays-on-Demand Gene Expression System, Applied Biosystems), CLOCK (Hs00231857_m1, Assays-on-Demand Gene Expression System, Applied Biosystems), PER1 (Hs00242988_m1, Assays-on-Demand Gene Expression System, Applied Biosystems), PER2 (Hs00256144_m1, Assays-on-Demand Gene Expression System, Applied Biosystems) and GAPDH (Hs99999905_m1, Assays-on-Demand Gene Expression System, Applied Biosystems) were used. All reactions were carried out in a 10 µl final volume containing 5 µl Master Mix, 0.5 µl of the specific assay and 4.5 µl of 1∶2 diluted cDNA. The amplification was performed under following conditions: 95°C for 10 min followed by 40 cycles of denaturation at 95°C for 15 s and annealing/elongation at 60°C for 1 min. Standard curves for all assays show similar gradients (coefficient of variation 6.8%, data not shown). CRY1 mRNA expression was normalized against the housekeeping gene glyceralaldehyde-3-phosphate dehydrogenase (GAPDH) as endogenous reference by computing the difference between the respective Ct values (ΔCt = C_t_[gene]−C_t_[*GAPDH*]). All PCR reactions were performed in duplicate (mean coefficient of variation for all target genes was below 1%). The mean threshold cycle number (*C*_t_) for each tested mRNA was used to quantify the relative expression of each gene: 2^−ΔCt^.

#### DNA methylation analysis of the CRY1 gene by bisulphite genomic sequencing

Bisulphite treatment of genomic DNA was carried out using the EpiTect Bisulphite Kit (Qiagen, Hilden, Germany) according to the manufacturer's instruction. The primers for amplifying bisulphite-modified DNA were: CRY1-forward: 5′-TTTGTGAGGGAAGGTTTAGTTT-3′, CRY1-reverse: 5′-AACAATTTCCAAACCCTCC-3′. For possible sequencing of the PCR product we attached a tag (forward 5′-CTTGCTTCCTGGCACGAG-3′, reverse 5′-CAGGAAACAGCTATGAC-3′). The PCR was carried out as follows: denaturation at 95°C for 10 minutes followed by 35 cycles comprising a second denaturation of 30 seconds at 94°C, annealing at 61°C for 30 seconds and extension at 72°C for 45 seconds, followed by 7 minutes elongation at 72°C. The sequence of the PCR product is depicted in Figure S1. PCR products were separated by agarose gel electrophoresis, excised and purified by gel extraction with MinElute Gel Extraction Kit (Qiagen, Hilden, Germany). PCR products were ligated into pGEM®-T Easy Vector (Promega, Madison, USA) and transformed in XL1-Blue Competent Cells (Stratagene, La Jolla, CA, USA). Plasmid DNA isolated from multiple colonies derived from each PCR product were sequenced using the CRY1 reverse primer on an ABI 3130 Genetic Analyzer.

#### DNA methylation analysis of the CRY1 gene by Bisulphite MassArray assays

Bisulphite treatment of genomic DNA was carried out using the EpiTect Bisulphite Kit (Qiagen, Hilden, Germany) according to the manufacturer's instruction. The reverse primer contained a T7-promoter tag for in vitro transcription (5′-cagtaatacgactcactatagggagaaggct-3′) and the forward primer was tagged with a 10 mer (5′-aggaagagag-3′). Bisulphite-treated DNA was PCR amplified (denaturation at 95°C for 10 minutes followed by 40 cycles comprising a second denaturation of 30 seconds at 95°C, annealing at 61°C for 30 seconds and extension at 74°C for 30 seconds, followed by 10 minutes elongation at 74°C), PCR products were purified by gel extraction with MinElute Gel Extraction Kit (Qiagen, Hilden, Germany). 5 µL of the PCR product were used for the assay. Bisulphite MassArray assays were performed by the Genomics Core Facility, Albert Einstein College of Medicine. The data were analyzed using the analytical pipeline previously published [17]. DNA quality and no-template controls, 0%, and 100% methylated DNA were included in all assays.

#### 50 K SNP array analysis of CRY1 copy number at chromosome 12q23

For SNP array studies, genomic DNA was extracted from CD19 positively selected PBMCs using the QIAamp blood kit (Qiagen, Hilden, Germany) following the manufacturer's instructions. Array experiments were performed according to the standard protocol for Affymetrix GeneChip Mapping 50 K arrays (Affymetrix). Briefly, a 250 ng sample of DNA was digested with XbaI, ligated to adaptors, amplified by PCR, fragmented with DNAse I, and biotin-labeled. The labeled samples were hybridized to the Affymetrix 50 K SNP XbaI mapping array (Affymetrix Inc., Santa Clara, CA) followed by washing, staining and scanning.

The acquired signal data was normalized with the dChip [18] program, using model-based expression, perfect match (PM)-only background subtraction and quantiles as probe-selection method. The normalized signal was then used as raw copy number per SNP and further analyzed by the GLAD algorithm [19] included in the GenePattern [20] suite, which segmented the data and assigned aggregated copy numbers to segments. A segment was defined as aberrant if its copy number was below 1.7 (loss) or above 2.3 (gain). The resulting list of aberrant regions was in addition filtered for regions consisting of more than 10 SNPs to exclude regions resulting from random noise in the copy number signal.

### Statistical analysis

Comparisons of clinical and biological parameters between subgroups were carried out using the Mann-Whitney-U test for continuous variables and Fisher's exact test for categorical data. Correlation between CRY1 delta CT and percentage of promoter methylation was tested using Spearman correlation. The Wilcoxon test for paired samples was used to compare CRY1 delta CT analysed in B and T cells from ND. Survival analysis was carried out with the Kaplan-Meier method and differences in treatment free survival between risk groups were tested with the log-rank test. All analyses were performed using R statistical software version 2.10.1 (R Development Core Team, Vienna, Austria, http://www.r-project.org) and GraphPad Prism Version 5.04 (GraphPad Software).

## Results

### Analysis of CRY1 expression in normal donor derived vs. CLL PBMC

**Figure 1:**
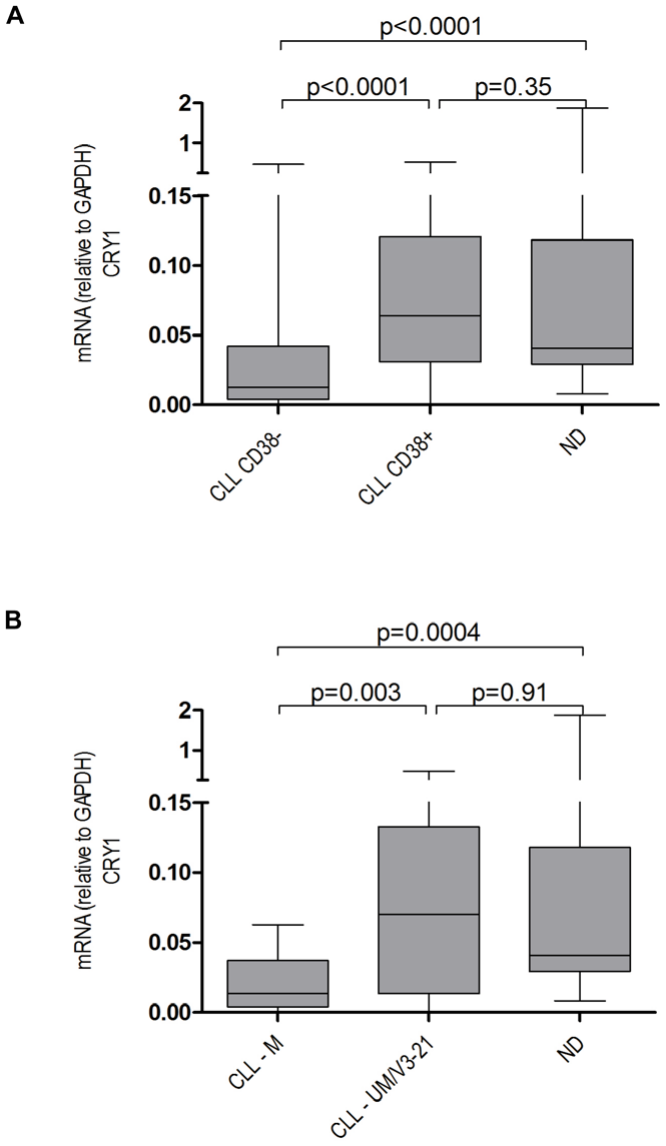
Expression of CRY1 in CLL subgroups and normal donors. *CRY1* mRNA expression in normal donors (ND, n = 35) in comparison to CLL samples from prognostic subgroups defined by CD38 expression (A, CD38+ samples, n = 36 vs. CD38− samples, n = 39) and IgVH mutational status (B, IgVH unmutated/V3-21, UM/V3-21, n = 23 vs. IgVH mutated, M, n = 18). mRNA levels are relative to GAPDH. Data are presented in a box-and-whisker format: the difference of the 25th and 75th percentile form the box (interquartile range, IQR), with the median marked as a line; the whiskers go down to the smallest value and up to the largest values. The Mann-Whitney U-test was used to compute p-values for pairwise comparisons.

We measured the mRNA expression of the circadian gene CRY1 in peripheral blood mononuclear cells (PBMC) containing more than 80% CD19+CD5+ leukemic B cells as determined by multiparameter flow cytometry in a cohort of 76 CLL patients (Table 1). In line with our previously published work [8], [9] we observed significantly higher CRY1 mRNA levels in high-risk(HR) patients defined by the expression of CD38 and/or unmutated IgVH genes as compared to their CD38 negative and/or IgVH mutated low risk (LR) counterparts (Figure 1A, 1B). We could also confirm the prognostic value of CRY1 expression in CLL by comparing the clinical outcome of CLL patients with high vs. low CRY1 expression using the median ΔCt value as a cut-off. Significant differences in treatment free survival (TFS) were observed between the two groups (Figure S2). Of note, comparative analysis with PBMC derived from age matched normal blood donors revealed that these expression differences can be attributed to an under-expression of CRY1 in LR CLL cases rather than over-expression in the HR group (Figure 1A, 1B). As the cellular composition of normal PBMC and PBMC isolated from CLL patients is known to vastly differ with regard to the content of B and T cells we compared mRNA expression levels of CRY1 in immunomagnetically purified CD19+ B cells and CD3+ T cells from normal donors (Figure S3). CRY1 expression was very similar in these normal lymphocyte subsets suggesting that under-expression in LR CLL samples relative to ND controls reflected true down-regulation of the gene in the leukemic cells rather than differences in the cellular composition of the PBMC samples.

### Dysregulation of CRY1 in CLL cannot be explained by chromosomal aberrations

Aiming at investigating the molecular mechanism(s) underlying disrupted CRY1 expression in CLL cells we then speculated that this phenomenon may be explained by aberration of this gene at chromosome 12q23–q24.1. Exploiting a panel of CLL cases which had been previously investigated for the presence of structural chromosomal abnormalities [21] by high resolution SNP array profiling we could not detect loss of chromosomal material at the CRY1 locus in any of the 55 individual CLL samples analyzed (data not shown). Moreover, an elevated copy number at this position is only detected for samples harbouring a trisomy 12, all other samples exhibit the regular two copies of this gene. The CLL patient cohort of this study (n = 76) comprises 12 cases with a trisomy 12. Among the CD38+ population 10 patients had a trisomy 12 whereas 14 were negative. Among the CD38− patients 2 had a trisomy 12 whereas 28 subjects proved to be negative. No differences in CRY1 mRNA expression were noted neither in the CD38+ nor the CD38− subgroups defined by the presence or absence of trisomy 12 (p = 0.54 and 0.49, respectively).

### DNA methylation analysis of the CRY1 gene

**Figure 2:**
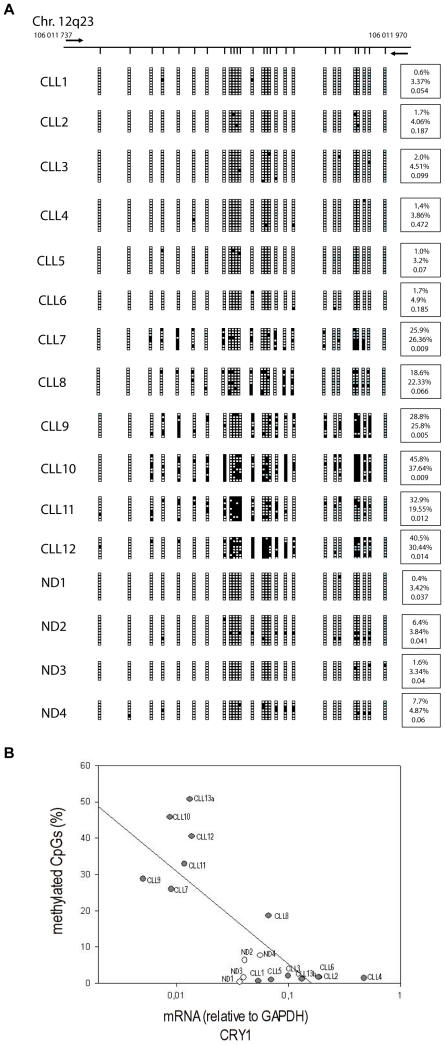
Analysis of CRY1 CpG island promoter methylation status measured by bisulphite genomic sequencing. **A** Results of bisulphite genomic sequencing performed on 6–12 clones of 4 normal donors (ND) and 12 different CLL samples. Top, schematic depiction of the analyzed area of the CRY1 CpG island around the transcription start site (horizontal black line). Short vertical lines represent CpG dinucleotides. The location of PCR primers are indicated as bold black horizontal arrows. Presence of methylated or unmethylated CpG dinucleotides is indicated by solid or open squares respectively, blue squares indicate not analyzable CpG dinucleotides. The mean standard error for all samples is 1.99 reflecting the low variability between the methylation profile of each analyzed clone. At the right hand side of each graph, first the percentage of methylated CpGs for each individual sample is depicted, below is the percentage of methylated CpGs measured by Bisulphite MassArray and at the bottom each sample's CRY1 mRNA expression relative to GAPDH is shown. **B** Samples from CLL patients and ND were subjected to both CRY1 mRNA expression and DNA methylation analysis by bisulphite genomic sequencing. mRNA expression values and percentage of methylated CpG were found to be highly correlated (r = −0.62, p = 0.006, Spearman correlation). The regression line in the plot was produced by linear regression analysis using promoter methylation as dependent and CRY1 mRNA expression as independent variable (open circles indicate ND).

**Figure 3:**
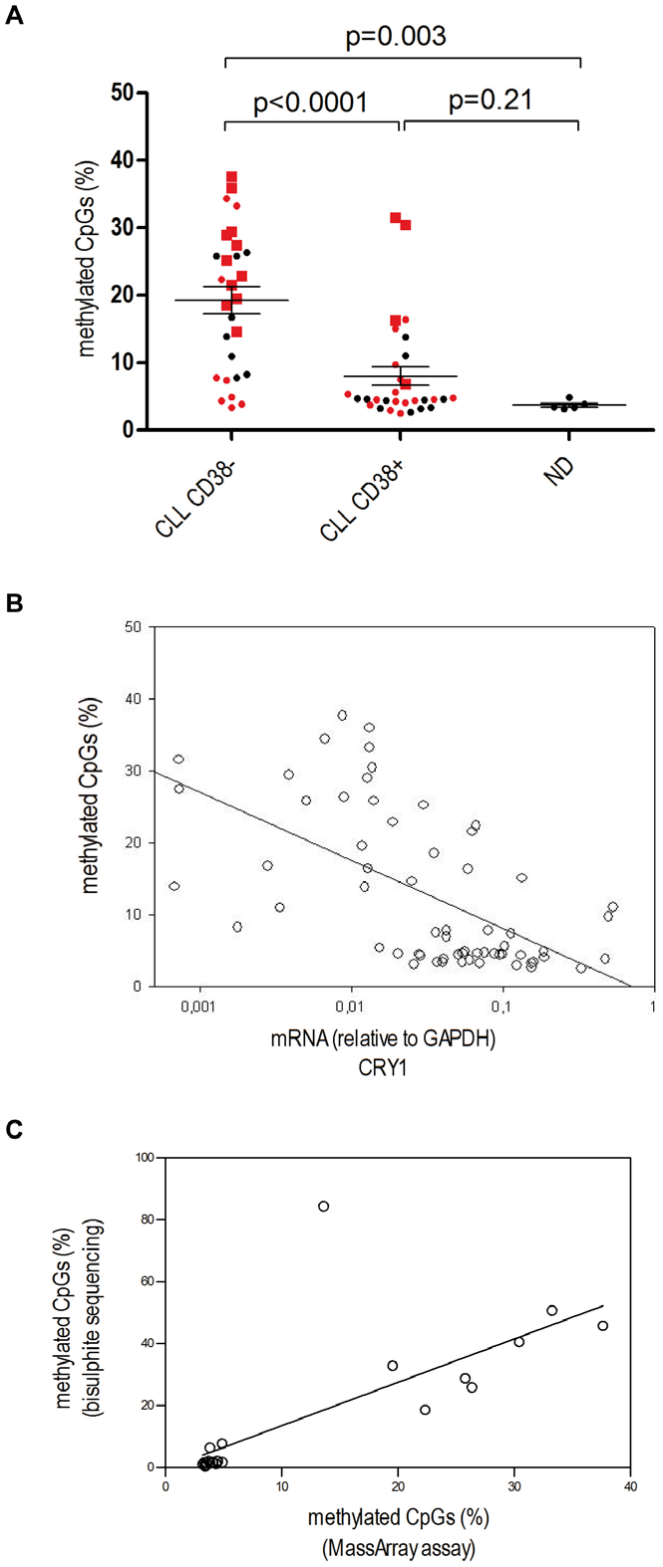
Analysis of CRY1 CpG island promoter methylation status measured with the Bisulphite MassArray assay. **A** Results of CRY1 CpG island methylation analysis performed on CLL samples and normal donors (ND) grouped by CD38 expression (CD38− samples, n = 28; CD38+ samples, n = 30; ND, n = 5). Each value represents the average amount of methylated CpGs of all analyzable CpGs within the CpG island promoter from one patient. The values represent the mean of duplicate experiments. The IgVH mutational status of each patient (if available) is highlighted in red; circles and squares indicate unmutated IgVH/V3-21 and mutated IgVH status, respectively. The median is marked as a line, error bars indicate SEM. Unpaired two-tailed t-test was used to compute p-values. **B** Samples from CLL patients and ND were subjected to both CRY1 mRNA expression and DNA methylation analysis with the bisulphite MassArray assay. mRNA expression values and percentage of methylated CpG were found to be highly correlated (r = −0.63, p<0.0001, Spearman correlation). The regression line in the plot was produced by linear regression analysis using promoter methylation as dependent and CRY1 mRNA expression as independent variable. **C** Correlation between the methylation data resulting from bisulphite genomic sequencing and the MassArray method showed high consistency (r = 0.86, p<0.0001, Spearman correlation).

As CRY1 has been previously shown to undergo aberrant DNA methylation events in various human cancers [7], [22], we then examined whether epigenetic silencing could explain CRY1 mRNA expression differences in CLL. DNA methylation was studied within the promoter region of the CRY1 gene (Figure S1) employing sequencing of cloned PCR products generated from bisulphite-modified DNA extracted from immunomagnetically purified CD19+ B cells from the peripheral blood of CLL (N = 14) and normal donors (N = 4). We observed that patients with low CRY1 expression show hypermethylation of the analyzed region in comparison to patients with a high CRY1 expression and normal donors (Figure 2A). Using the Sequenom MassArray Compact System for methylation analysis in the same CRY1 promoter region we then confirmed these results in a cohort of 58 CLL samples and 5 normal donors (Figures 3A and S4, Table S3). LR CLL patients either defined by CD38 or IgVH mutational status showed a significantly higher degree of methylation of the CRY1 promoter in comparison to the HR group. In all, there was a statistically highly significant inverse correlation between the percentage of methylated CpGs and CRY1 mRNA expression as detected by qRT-PCR (Figure 2B and 3B). Comparing both methods for methylation analysis a high correlation (r = 0.89, p<0.0001) proves the consistency and robustness of each of the applied methods (Figure 3C).

### Prognostic value of CRY1 methylation pattern in CLL

**Figure 4:**
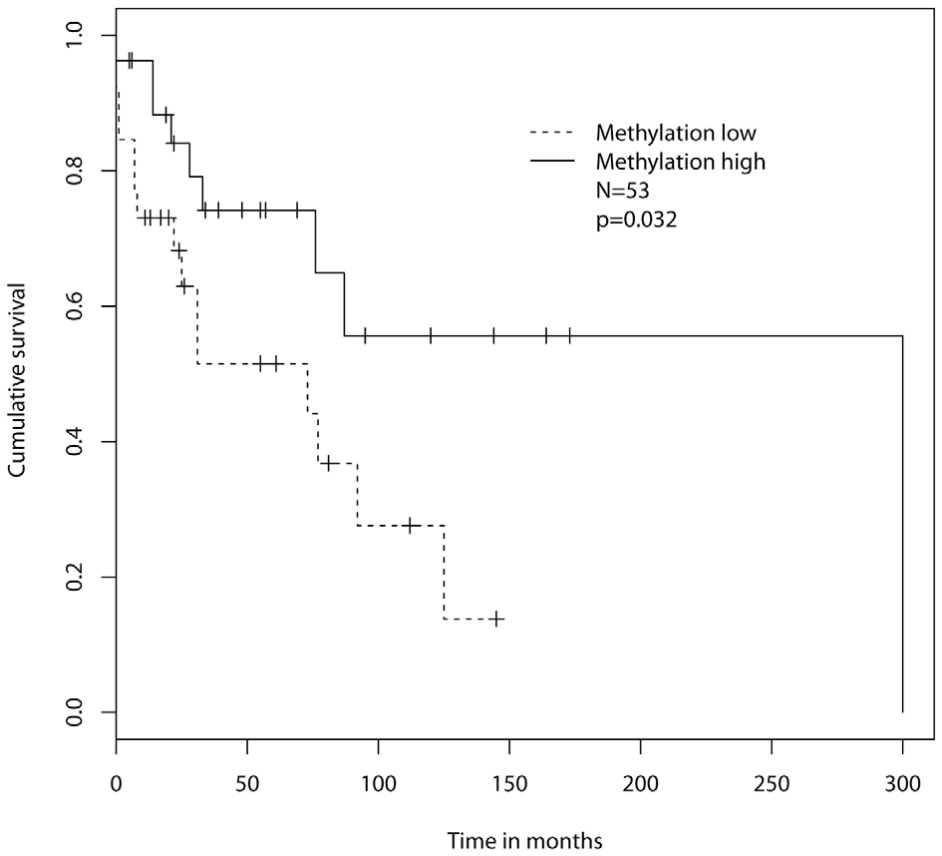
Treatment-free survival according to the methylation status of the CRY1 promoter region. Treatment-free survival of 53 CLL patients with lowly methylated (n = 26) and highly methylated (n = 27) CRY1 promoter.

To assess the prognostic value of the methylation profile of the CRY1 promoter we defined two groups, i.e. patients with lowly methylated and highly methylated promoter region. The threshold value of 9% was derived from the median value of the CLL samples subjected to MassArray methylation analysis. On survival analysis patients with a highly methylated CRY1 promoter region exhibited a significantly longer treatment-free survival as compared to the hypomethylated patient subgroup (Figure 4).

### Analysis of CRY1 expression over time

Twenty-seven patients were studied at two or more time points using the qRT-PCR assay. As illustrated in Figure S5, CRY1 mRNA expression was relatively stable over time in the majority of patients with stable disease (10/13 (77%), Figure S5, left panel) as well as in patients with progressive disease (12/14 (86%), right panel). The fact that three of the five patients with increasing CRY1 mRNA levels had a comparably benign course of disease may suggest that changes in CRY1 expression over time are not stringently related to disease progression. Moreover, in 6 of the 14 patients (marked by arrows in Figure S5) with a progressive course of the disease first treatment was initiated after collection of the baseline sample in this longitudinal analysis. Although all these patients responded to therapy, no reduction in CRY1 mRNA expression could be documented. It is noteworthy that we did not observe a single case with decreasing CRY1 mRNA levels during follow-up in the entire cohort of patients with samples available for sequential analysis.

Interestingly, one patient with a low baseline CRY1 expression and a correspondingly high degree of CpG island methylation experienced disease progression as evidenced by rising lymphocyte counts and transition from Binet stage A to stage C disease. Increasing CRY1 expression in this particular patient was shown to be associated with loss of CRY1 promoter hypermethylation (Figure S6).

### Analysis of the role of CRY1 within the circadian clock's transcription/translation-based feedback loop in CLL

At the molecular level, circadian rhythms are encoded by an autoregulatory loop composed of a set of transcription activators (CLOCK/BMAL1) that induce expression of PER and CRY. Accumulated PER and CRY proteins in turn inhibit BMAL1/CLOCK transcriptional activity and repress their expression. Thus, aberrant silencing of CRY1 expression in LR CLL may be associated with dysregulated expression of other circadian genes. Indeed, we could observe increased PER2 and CLOCK mRNA levels in CLL as compared to ND derived PBMC, whereas mRNA levels of BMAL1 and PER1 were similar in both groups (Figure S7). However, changes in PER2 and CLOCK expression were noted in both LR and HR CLL samples and could not be related to alterations in CRY1 expression (Figure S7) suggesting that these abnormalities may occur independently of each other.

### Analysis of CRY1 expression and CpG island hypermethylation in other hematologic malignancies

**Figure 5:**
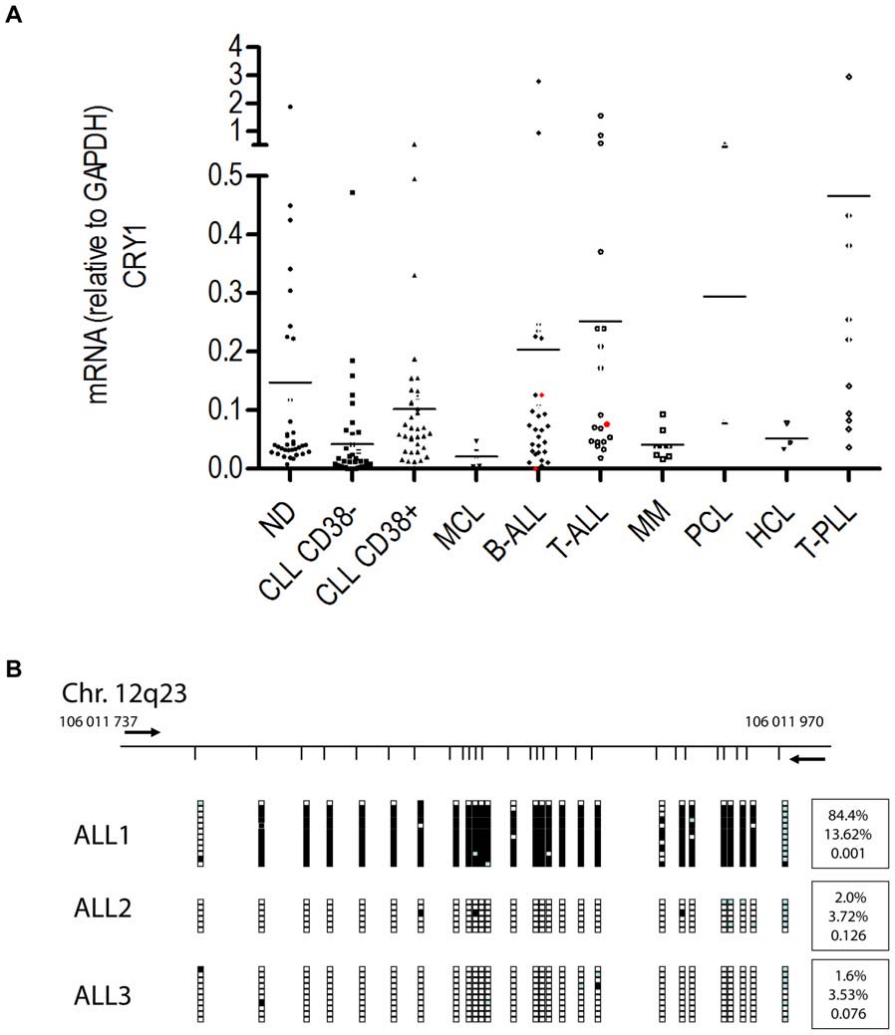
Comparative analysis of CRY1 expression in a panel of different lymphoid malignancies. qRT-PCR analyses of PBMC samples obtained from patients with T-prolymphocytic leukemia (T-PLL), mantle cell lymphoma (MCL), plasma cell leukemia (PCL), hairy cell leukemia (HCL), B and T cell acute lymphoblastic leukemia (B-ALL, T-ALL), CLL and normal donors (ND), A. Red characters indicate samples that were further subjected to DNA methylation analysis of the CRY1 promoter, A. Analysis of CRY1 CpG island promoter methylation status, B. For experimental details and description of the graph in panel B refer to the legend of Figure 2.

From a cancer biology standpoint we next wanted to test whether deregulated CRY1 expression may also be observed in other lymphoid malignancies than CLL. To this end we employed the CRY1 qRT-PCR assay to screen RNA samples isolated from a wide range of lymphoproliferative disorders including T prolymphocytic leukemia (T-PLL, n = 10), mantle cell lymphoma (MCL, n = 6), hairy cell leukemia (HCL, n = 3), multiple myeloma (MM, n = 8), plasma cell leukemia (PCL, n = 2) and B and T lineage acute lymphoid leukemia (n = 29 resp. n = 19). In disease entities with more than 5 observations, statistical comparisons with ND derived PBMC did not reveal significant expression differences (Figure 5A). However, this finding needs to be interpreted with caution because of the small numbers of samples tested in this series. The availability of a comparably large number of ALL samples (n = 48) allowed for a more detailed analysis of this disease group. The clinical characteristics of this patient cohort are given in Table S2. Similar to its distribution in CLL, CRY1 expression in ALL was found to be very heterogeneous where one patient with a mature B-ALL exhibited a markedly low CRY1 transcript level (Figure 5A). This particular sample demonstrated a high degree of CRY1 promoter methylation while two other ALL samples with a higher CRY1 expression were nearly completely demethylated (Figure 5B).

Next we investigated the prognostic value of CRY1 expression in ALL. To this end we compared the clinical outcome of ALL patients with high vs. low CRY1 expression using the median ΔCt value as a cut-off. No significant differences in overall survival (OS) were observed between the two groups (Figure S8).

## Discussion

In this study, we analyzed the expression of CRY1 mRNA, which encodes a key component of the central and peripheral circadian oscillator, in the PBMC from normal blood donors and patients with CLL [8], [11], [23], [24]. In line with our previous work [8], [9] and that of others [10], [11] we detected elevated CRY1 transcript levels in patients with high risk disease defined by the expression of CD38 and/or unmutated IgVH (UM) genes as compared to their CD38 negative and/or IgVH mutated (M) low risk (LR) counterparts. Lewintre et al. confirmed these results in a recently published microarray-based gene expression profiling study including 36 patients with early-stage CLL [11]. Therefore, determination of CRY1 may have potential as a novel prognostic marker in CLL and should be tested in comparison to other established molecular risk factors in the setting of prospective randomized trials.

We now uncover that HR CLL cases and ND derived B cells exhibit comparable levels of CRY1 mRNA expression. Thus, disrupted CRY1 expression in CLL can be attributed to down-modulation of CRY1 in LR CLL cases rather than over-expression in the HR group. We then aimed to investigate the molecular mechanisms underlying down-regulation of CRY1 in LR CLL. As CRY1 had been previously shown to undergo aberrant DNA methylation events in various solid human malignancies including breast and ovarian cancers [3], [7], [22], we examined whether epigenetic silencing could also explain the observed CRY1 mRNA expression differences in CLL subgroups. To this end we performed comparative DNA methylation analysis of highly purified CD19+ B cells from the peripheral blood of 57 CLL patients and normal donors. Indeed, our results show that CRY1 is transcriptionally silenced by promoter hypermethylation in LR CLL cases while HR cases and ND derived B cells exhibit hypomethylated CRY1 promoter regions. Importantly, DNA methylation analysis was complemented by qRT-PCR performed on the same samples revealing a statistically highly significant inverse correlation between the percentage of methylated CpGs and CRY1 mRNA expression in individual cases, suggesting a direct regulation of CRY1 expression through methylation of its promoter. We could show that the methylation status of the CRY1 promoter predicts clinical outcome in CLL patients; where aberrant hypermethylation was associated with a more benign course of the disease. To date, only few reports describe an aberrant methylation phenotype as a predictor of outcome in haematological diseases. Recently, Irving et al reported a panel of methylation markers (CD38, HOXA4, BTG4) in which an overall methylation score was significantly associated with time to first treatment in CLL [25]. Olk-Batz et al [26] described that a high methylation profile is associated with an aggressive biological variant of juvenile myelomonocytic leukemia.

As stability of CRY1 expression by the leukemic cell clone over time is an important prerequisite for its reliable use as a prognostic marker [27], we sequentially analyzed CRY1 expression in 27 patients. CRY1 transcript levels were remarkably stable in the majority of patients and no consistent changes were noted in relation to alterations in disease activity and treatment history of individual patients. Significant expression changes during follow-up occurred in 19% of the patients, which could limit the value of this marker under routine clinical conditions. However, these findings are limited by the comparably small number of CLL cases analyzed in this study and thus need to be validated in a larger patient cohort. In one patient with a low baseline CRY1 expression and a correspondingly high degree of CpG island methylation disease progression was linked to increasing CRY1 expression and hypomethylation of the CRY1 promoter region. In aggregate, these findings raise the possibility that epigenetic silencing of CRY1 occurs early in the disease and may be lost during disease progression at least in rare cases. Conversely, it appears unlikely that the leukemic cells acquire epigenetic silencing of CRY1 during the course of the disease as none of the 27 patients in the longitudinal analysis showed a decrease of CRY1 expression. It would be interesting to further investigate this hypothesis by systematically comparing the prevalence of CRY1 methylation events in individuals with monoclonal B cell lymphocytosis with Binet stage A, B and C CLL patients.

While differential CRY1 expression in HR vs. LR CLL subgroups is now well established [8]–[11], [28], the functional consequences of CRY1 down-modulation in the leukemic cells are currently unknown. It is tempting to speculate that epigenetic silencing of CRY1 may contribute to the benign clinical behaviour of LR CLL cases. At first sight this notion appears counterintuitive to the general conception that the core components of the molecular clock machinery may function as tumor suppressor genes [29]–[32]. This view is mainly based on epidemiological and genetic evidence indicating that disruption of circadian rhythms might be directly linked to cancer development including non-Hodgkin-lymphoma [2], [5], [33]–[35]. While the circadian genes PER1 and PER2 have been clearly shown to function as tumor suppressors in the mouse model [30], a recent study showed that epidermal deletion of BMAL1 in a transgenic mouse model which spontaneously develops squamous tumours leads to significantly fewer neoplastic lesions [36]. Along the same line transgenic CRY deficient mice do not show a predisposition to cancer [6]. Furthermore and somewhat unexpectedly, ablation in the mouse of both CRY genes in a TP53^−/−^ background delays the onset of cancer [13]. This latter observation supports our hypothesis that epigenetic silencing of CRY1 may functionally contribute to the indolent clinical behaviour of LR CLL. It is currently unclear whether abrogation of one CRY gene, i.e. CRY1 or CRY2 suffices to fully block the circadian rhythmicity of an individual cell. Studies comparing the biologic characteristics of transgenic mice lacking either one or both CRY genes indicate a certain degree of functional redundancy [37].

Another matter of controversy is whether circadian rhythmicity per se or only certain core clock components are involved in tumorigenesis [4]. To address this issue we correlated CRY1 mRNA expression with other components of the circadian clock's transcription/translation-based feedback loop in individual CLL samples. Recently, a number of in vitro and animal studies have further elucidated the important functional role of CRY proteins for the regulation of the molecular clock. For example, work by Ye et al [38] demonstrated that CRY directly interacts with the BMAL1:CLOCK:E-box complex independent of PER resulting in inhibition of the transactivator function of CLOCK:BMAL1. Furthermore, Busino et al. [39] showed in an in vitro model that Cry1^−/−^ Cry2^−/−^ mouse embryonic fibroblasts are characterized by a loss of oscillation in Per1 and Per2 and exhibit increased Per2 but not Per1 mRNA transcript levels in comparison to control fibroblasts isolated from CRY wild type animals.

Here, we found that the expression of PER2 and CLOCK but not BMAL1 and PER1 are also disrupted in CLL as compared to ND derived control samples. However, contrary to what could be expected from above described findings [38], [39] these abnormalities could neither be correlated with changes in CRY1 expression nor the clinical course of the disease in individual CLL cases. In aggregate, these latter results clearly suggest that defects in the circadian molecular machinery may be a common phenomenon in CLL cells and are not restricted to CRY1. Ongoing work in our laboratory is aimed at determining the molecular and cellular consequences of silencing different circadian genes in CLL cells in vitro using siRNA oligonucleotide technology.

Finally, we wanted to determine whether CRY1 expression may also be disturbed in other lymphoid malignancies than CLL. To this end we measured CRY1 transcript levels in PBMC samples from a range of different disease entities including T-PLL, MCL, HCL, MM, PCL and B and T cell ALL. In a subgroup analysis focussing on ALL we found a heterogeneous expression pattern comparable to that observed in CLL. One patient with a mature B-ALL exhibited a particularly low CRY1 expression which correlated with a high degree of promoter methylation, suggesting that aberrant epigenetic silencing of this gene may also occur in other lymphoid disease entities. However, contrary to CLL survival analysis did not show a significant difference between ALL patients with high and low expression of CRY1, respectively.

In conclusion, our data indicate that the previously reported CRY1 gene expression differences in LR vs. HR CLL patients [8], [11] are caused by aberrant methylation of the CRY1 promoter in the LR patient subgroup. To our knowledge this is the first report in CLL research linking epigenetic silencing of a specific gene to an indolent clinical course of the disease.

## Supporting Information

Figure S1 **Map of CRY1 located on chromosome 12q23.3.** The chromosomal location and nucleotide sequence of CRY1 on chromosome 12q23.3 (indicated by the red vertical line) extending from 105,909,272–106,011,728. CRY1 has one CpG island consisting of 130 CpGs depicted as the green box (106,010,743–106,011,941). Methylation analyses were performed within this CpG island from 106,011,737–106,011,970. Red letters indicate the primer sequences used for DNA methylation studies.
(TIF)

Figure S2 **Kaplan-Meier curves for treatment-free survival in CLL according to high and low expression of CRY1.** The median CRY1 ΔCT value was used as a cut-off to define patient subgroups with high and low CRY1 mRNA transcript levels. Figure S2 illustrates the treatment-free survival in 73 CLL patients with high/low CRY1 mRNA levels. Statistical differences were analyzed by log-rank test.
(TIF)

Figure S3 **Comparative analysis of CRY1 expression in normal B and T cells.** qRT-PCR analysis of immunomagnetically purified CD19+ B vs. CD3+ T cells obtained from five normal donors. Statistical comparisons were performed using the Wilcoxon test for paired samples.
(TIF)

Figure S4 **Detailed results of CRY1 CpG island promoter methylation measured with Bisulphite MassArray assays.** Results of CRY1 CpG island methylation analysis performed on further 58 CLL samples subdivided by CD38 expression (CD38− samples, n = 28, CD38+ samples, n = 30, ND, n = 5). Each row represents the methylation profile of one individual patient. Putative fragmentation patterns are shown for T -cleavage reaction on the plus strand of an amplicon of the human genome. CG dinucleotides (filled circles) are numbered and color-coded according to their ability to be assayed, where gray indicates that the CG is located on a fragment whose molecular weight is outside the usable mass window, red indicates a molecular weight overlap with another fragment and blue indicates a uniquely assayable site. Fragmentation patterns are shown in corresponding colors, yellow highlights primer sequences. Methylation data are shown as an average from duplicates. Bar height denotes percent methylation on a scale from 0% (low) to 100% (high), error bars indicate median absolute deviation. CG sites that are putatively outside the usable mass window are indicated as boxes with gray background.
(TIF)

Figure S5 **Analysis of CRY1 expression over time.** In all, 27 patients were analyzed for CRY1 expression using qRT-PCR at two or more time points. Each line represents an individual patient. The left graph depicts 13 patients with a stable course of disease, on the right 14 patients with progressive disease are shown. Three patients indicated with dashed lines had a progressive disease but were not treated (right graph). Those patients receiving first line chemotherapy after collection of the first sample (baseline) are highlighted by individual colors whereas the time point of first therapy is indicated by vertical arrows. mRNA expression values are normalized to each patient's baseline, which is set to 1.
(TIF)

Figure S6 **Time course analysis of CRY1 mRNA expression and CpG island promoter methylation in patient #13 showing disease progression during follow-up.** Methylation analyses in panel B correspond to samples obtained at baseline (a) and after 24 months of follow-up (b). For experimental details and description of the graph in panel B refer to the legend of Figure 2.
(TIF)

Figure S7 **Analysis of circadian gene transcript levels in CLL cells by qRT-PCR.** Figure S7 shows PER1, PER2, CLOCK and BMAL1 expression in normal donors (ND, n = 24) in comparison to CLL samples from prognostic subgroups defined by CD38 expression (CD38+samples, n = 24 vs. CD38− samples, n = 27). mRNA expression is relative to GAPDH. Data are presented in a box-and-whisker format: the difference of the 25th and 75th percentile form the box (interquartile range, IQR), with the median marked as a line; the whiskers go down to the smallest value and up to the largest values. The Mann-Whitney U-test was used to compute p-values for pairwise comparisons.
(TIF)

Figure S8 **Kaplan-Meier curves for overall survival in ALL according to high and low expression of CRY1.** The median CRY1 ΔCT value was used as a cut-off to define patient subgroups with high and low CRY1 mRNA transcript levels. Figure S8 illustrates the overall survival for 48 patients with ALL according to high and low expression of CRY1 mRNA levels. Statistical differences were analyzed by log-rank test.
(TIF)

Table S1 **Characteristics of CLL patients tested for CRY1 promoter hypermethylation.**
(DOCX)

Table S2 **Clinical characteristics of ALL patients.**
(DOCX)

Table S3 **Individual methylation profile (MassArray assay) and CRY1 expression values.**
(DOCX)
