# Supplementary figures and images for: Epigenetic Silencing of the Circadian Clock Gene CRY1 is Associated with an Indolent Clinical Course in Chronic Lymphocytic Leukemia
Source: PLoS One. 2012 Mar 28;7(3):e34347. doi: 10.1371/journal.pone.0034347 (PMC3314606; doi:10.1371/journal.pone.0034347)

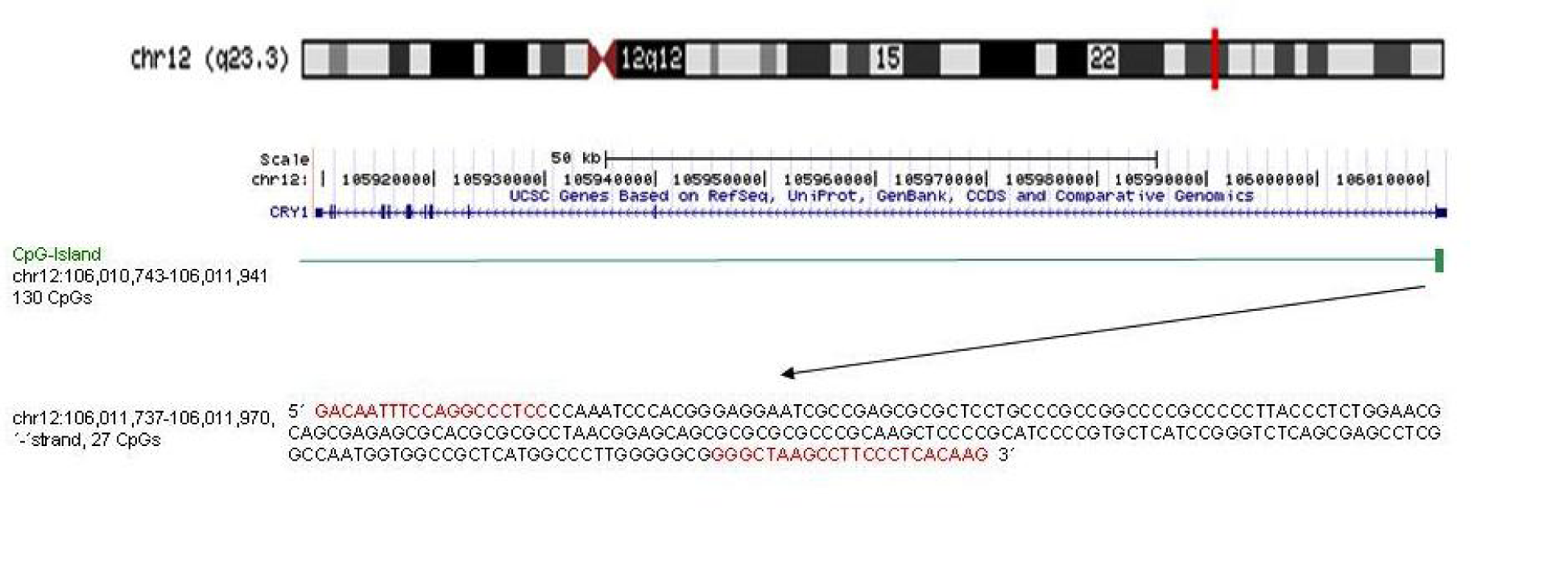

Supplement: Figure S1 — Map of CRY1 located on chromosome 12q23.3. The chromosomal location and nucleotide sequence of CRY1 on chromosome 12q23.3 (indicated by the red vertical line) extending from 105,909,272–106,011,728. CRY1 has one CpG island consisting of 130 CpGs depicted as the green box (106,010,743–106,011,941). Methylation analyses were performed within this CpG island from 106,011,737–106,011,970. Red letters indicate the primer sequences used for DNA methylation studies. (TIF) [file pone.0034347.s001.tif]

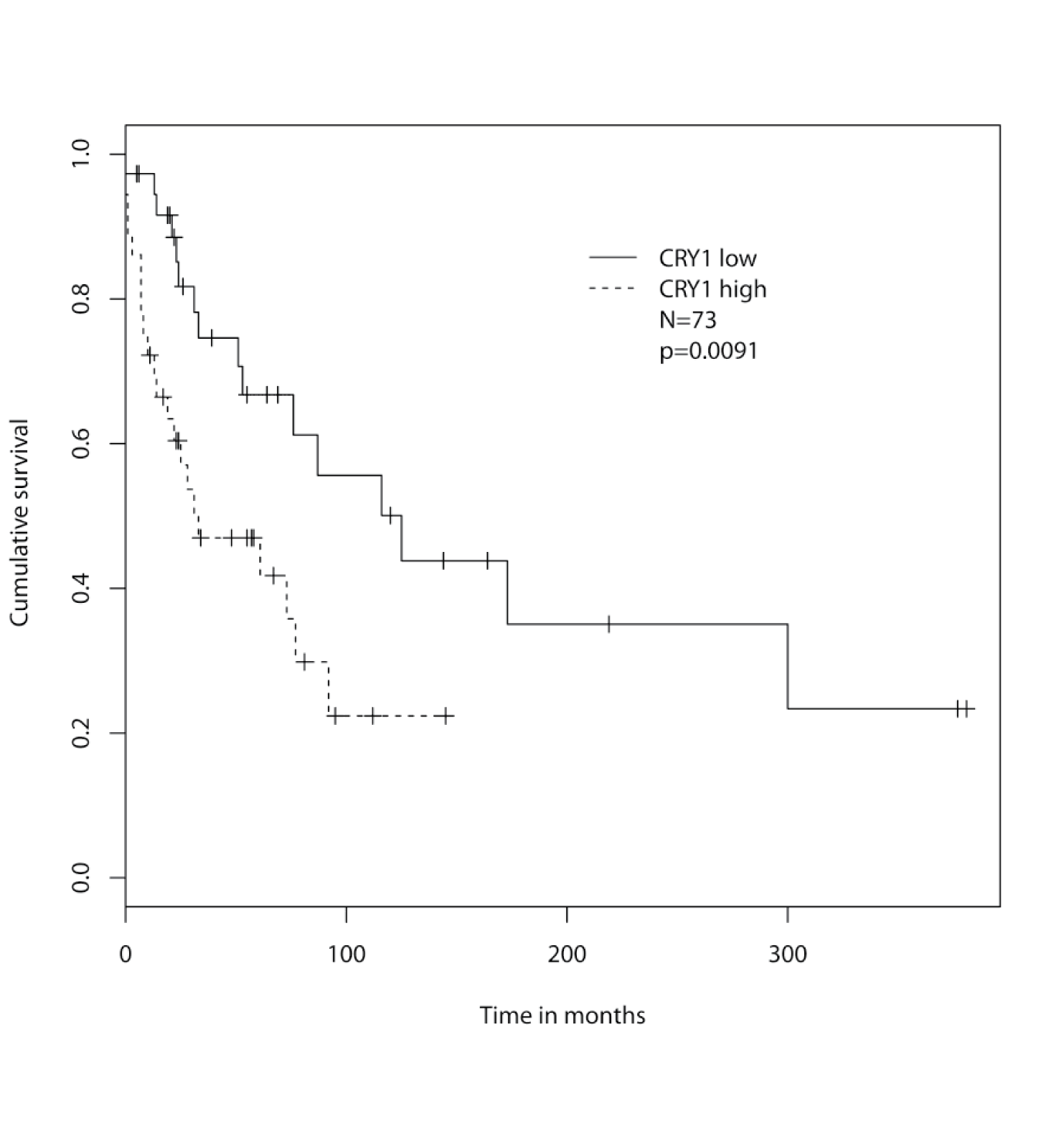

Supplement: Figure S2 — Kaplan-Meier curves for treatment-free survival in CLL according to high and low expression of CRY1. The median CRY1 ΔCT value was used as a cut-off to define patient subgroups with high and low CRY1 mRNA transcript levels. Figure S2 illustrates the treatment-free survival in 73 CLL patients with high/low CRY1 mRNA levels. Statistical differences were analyzed by log-rank test. (TIF) [file pone.0034347.s002.tif]

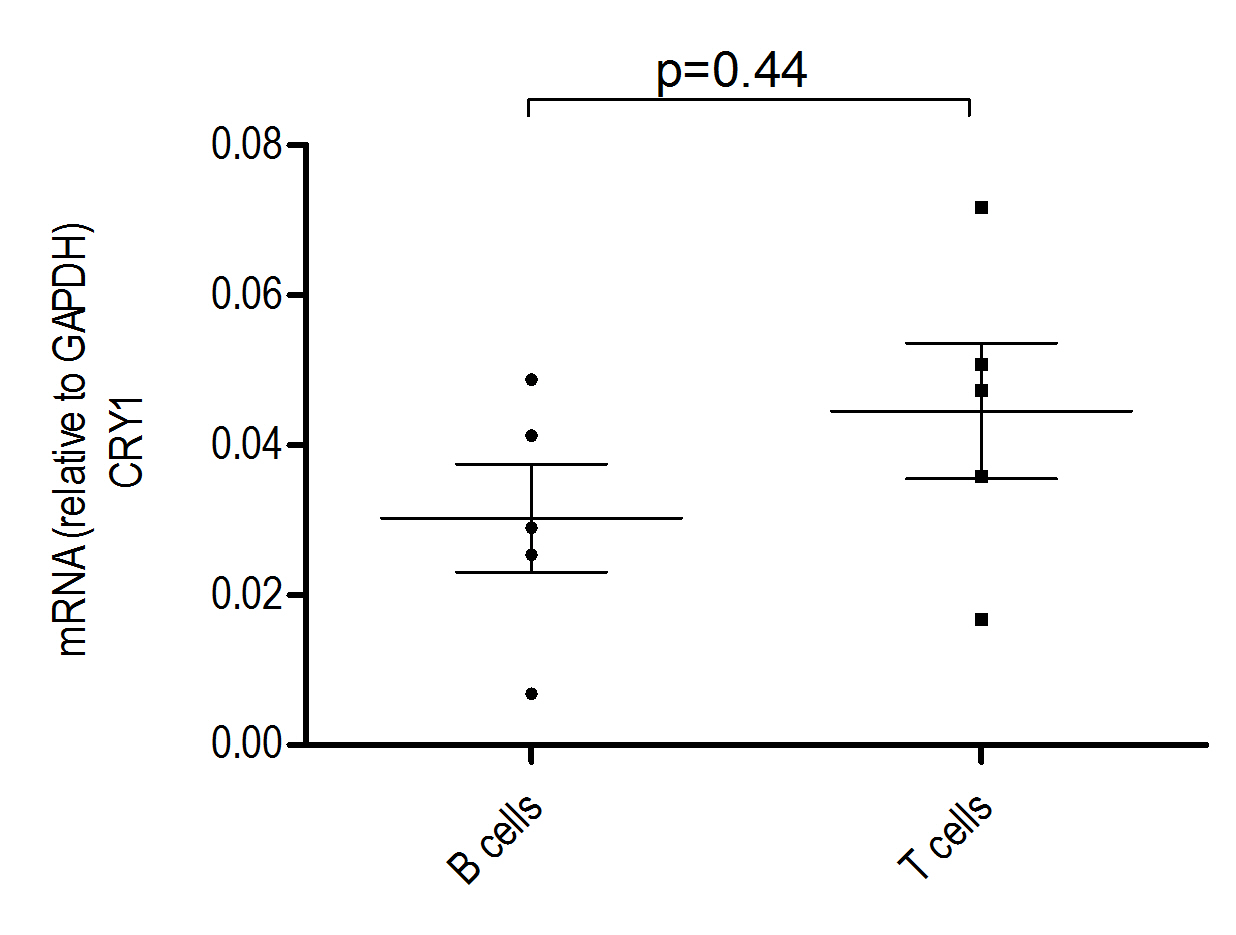

Supplement: Figure S3 — Comparative analysis of CRY1 expression in normal B and T cells. qRT-PCR analysis of immunomagnetically purified CD19+ B vs. CD3+ T cells obtained from five normal donors. Statistical comparisons were performed using the Wilcoxon test for paired samples. (TIF) [file pone.0034347.s003.tif]

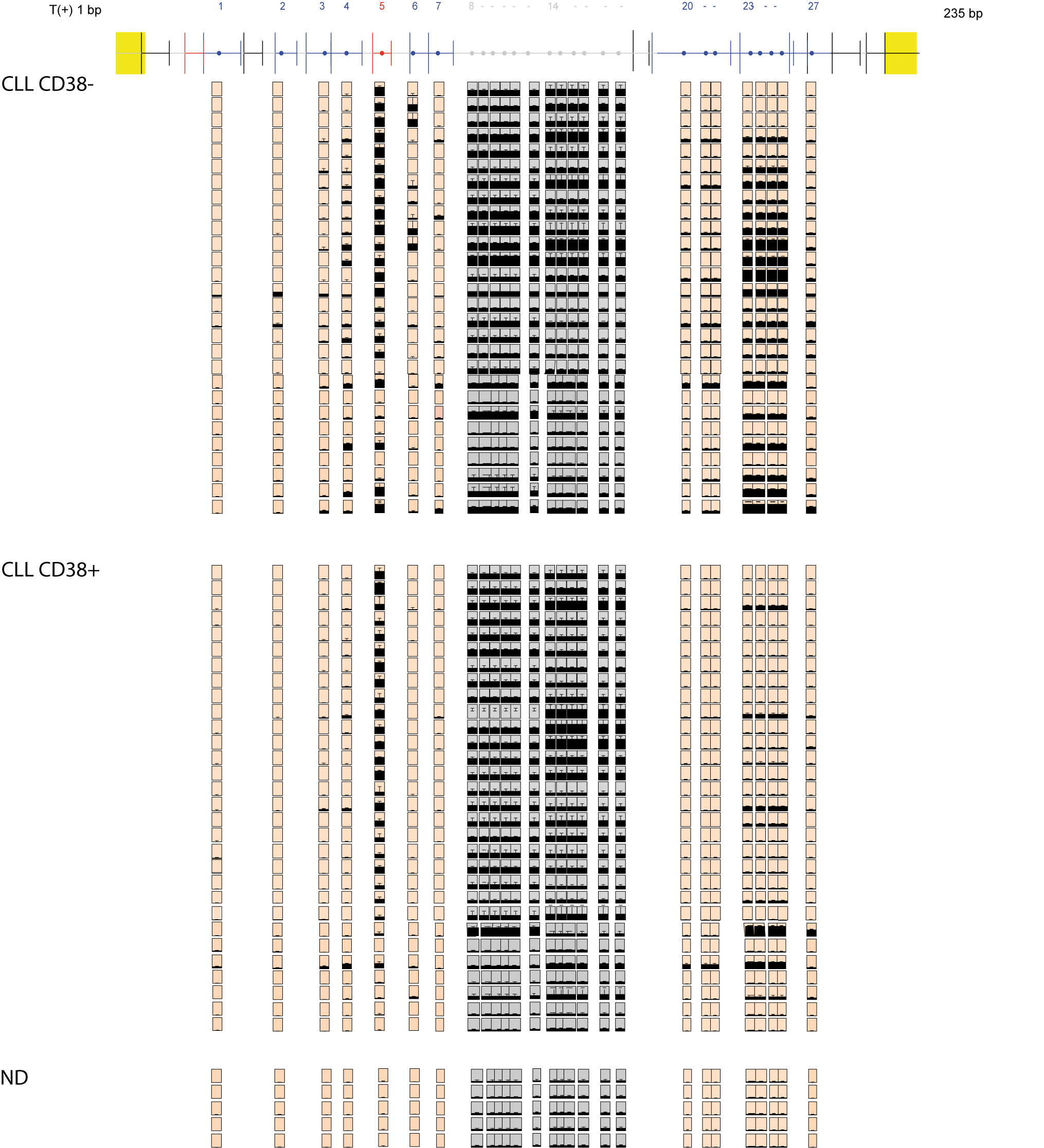

Supplement: Figure S4 — Detailed results of CRY1 CpG island promoter methylation measured with Bisulphite MassArray assays. Results of CRY1 CpG island methylation analysis performed on further 58 CLL samples subdivided by CD38 expression (CD38− samples, n = 28, CD38+ samples, n = 30, ND, n = 5). Each row represents the methylation profile of one individual patient. Putative fragmentation patterns are shown for T -cleavage reaction on the plus strand of an amplicon of the human genome. CG dinucleotides (filled circles) are numbered and color-coded according to their ability to be assayed, where gray indicates that the CG is located on a fragment whose molecular weight is outside the usable mass window, red indicates a molecular weight overlap with another fragment and blue indicates a uniquely assayable site. Fragmentation patterns are shown in corresponding colors, yellow highlights primer sequences. Methylation data are shown as an average from duplicates. Bar height denotes percent methylation on a scale from 0% (low) to 100% (high), error bars indicate median absolute deviation. CG sites that are putatively outside the usable mass window are indicated as boxes with gray background. (TIF) [file pone.0034347.s004.tif]

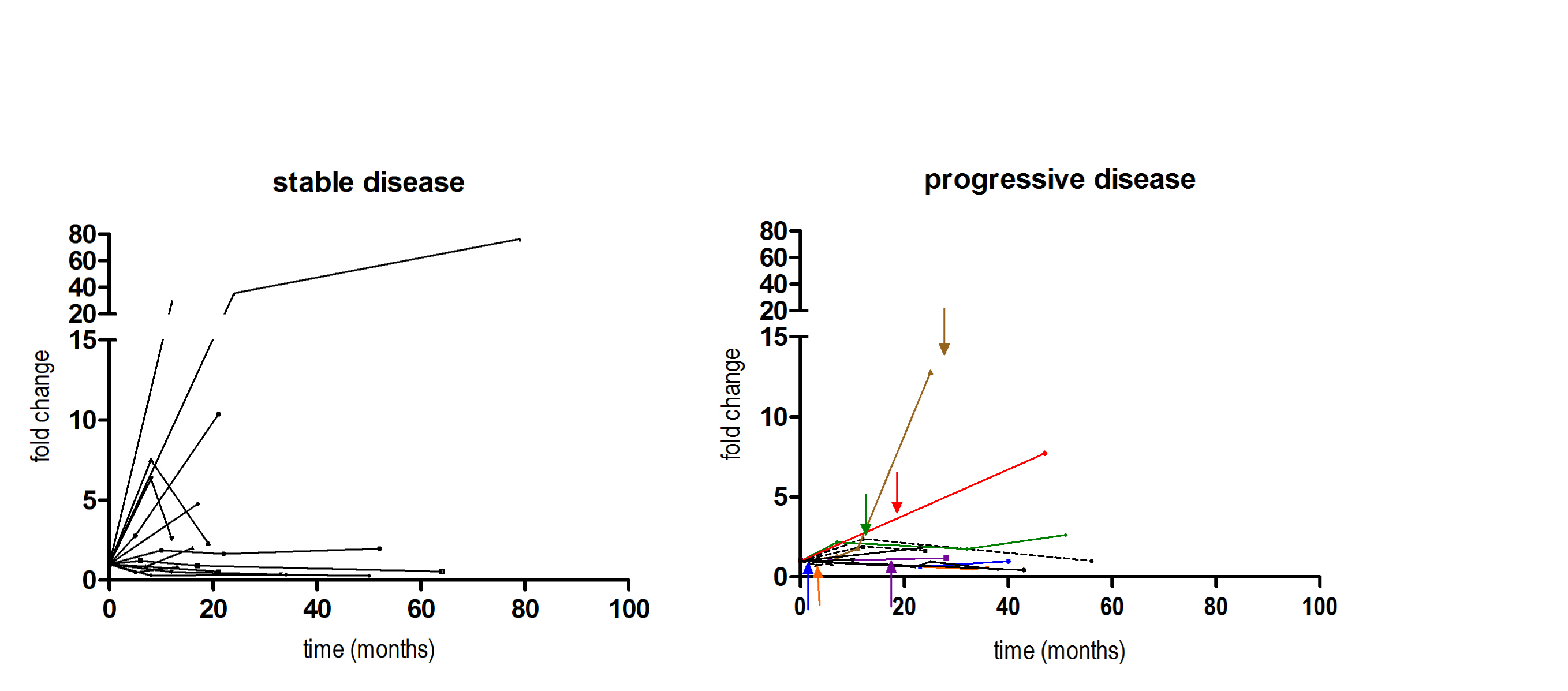

Supplement: Figure S5 — Analysis of CRY1 expression over time. In all, 27 patients were analyzed for CRY1 expression using qRT-PCR at two or more time points. Each line represents an individual patient. The left graph depicts 13 patients with a stable course of disease, on the right 14 patients with progressive disease are shown. Three patients indicated with dashed lines had a progressive disease but were not treated (right graph). Those patients receiving first line chemotherapy after collection of the first sample (baseline) are highlighted by individual colors whereas the time point of first therapy is indicated by vertical arrows. mRNA expression values are normalized to each patient's baseline, which is set to 1. (TIF) [file pone.0034347.s005.tif]

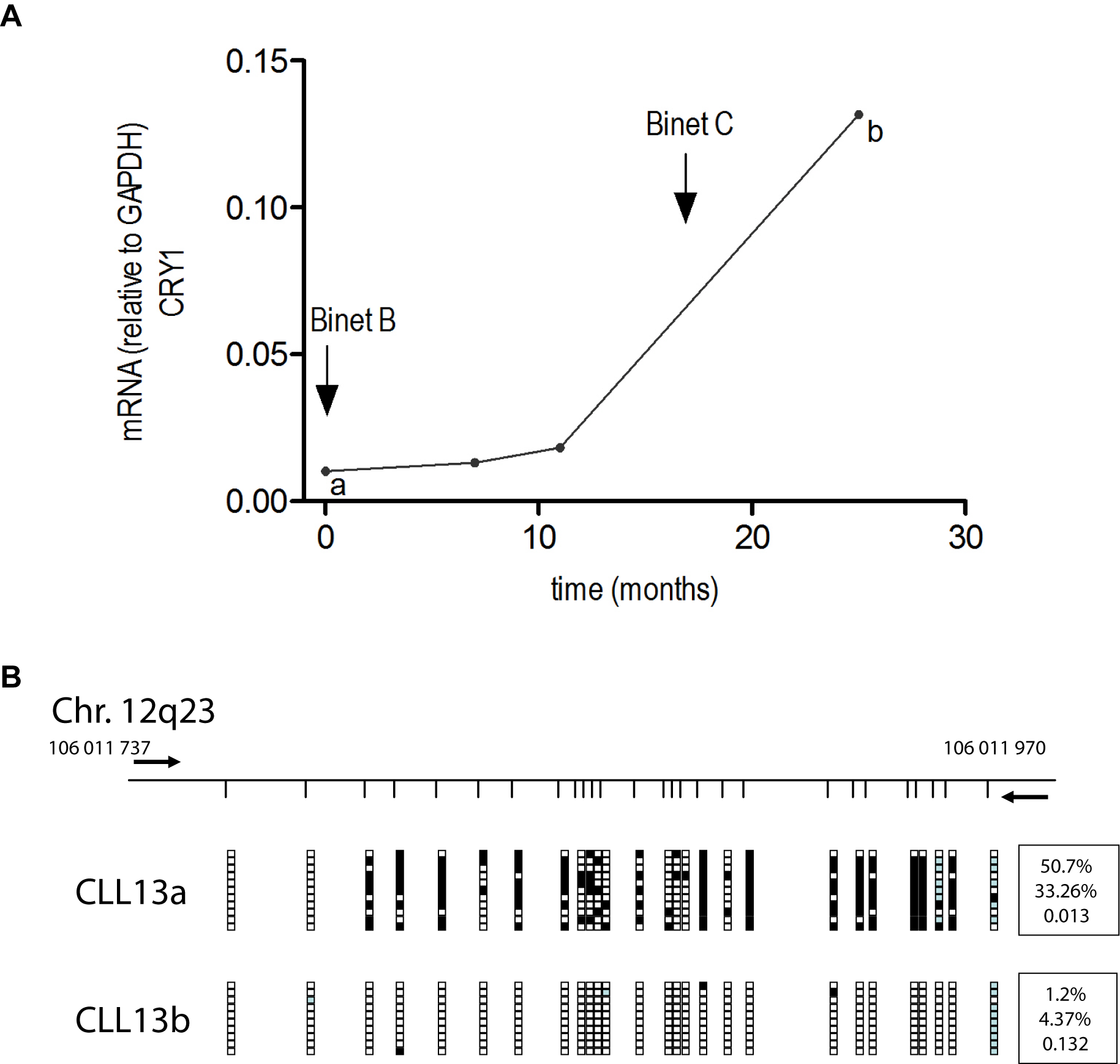

Supplement: Figure S6 — Time course analysis of CRY1 mRNA expression and CpG island promoter methylation in patient #13 showing disease progression during follow-up. Methylation analyses in panel B correspond to samples obtained at baseline (a) and after 24 months of follow-up (b). For experimental details and description of the graph in panel B refer to the legend of Figure 2. (TIF) [file pone.0034347.s006.tif]

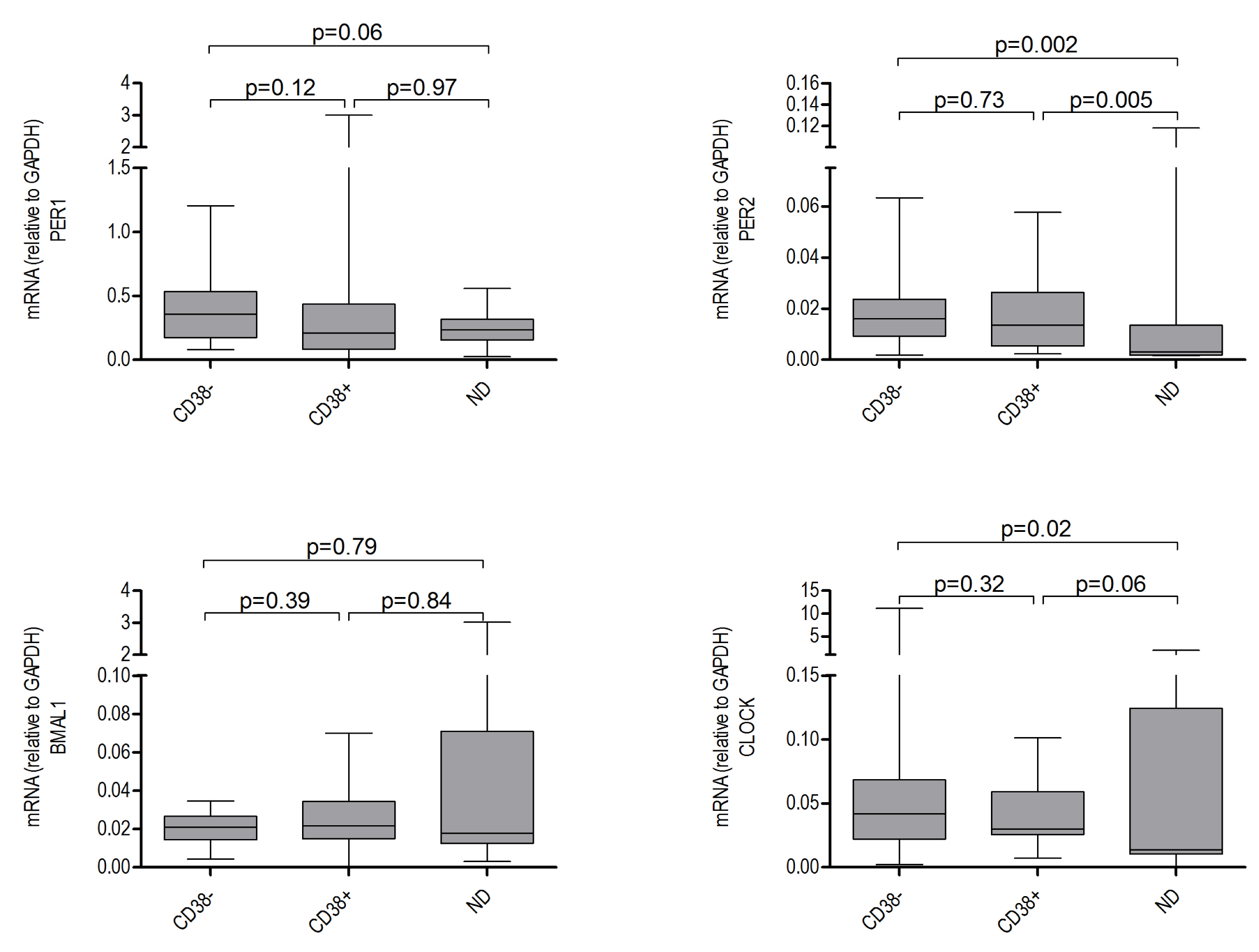

Supplement: Figure S7 — Analysis of circadian gene transcript levels in CLL cells by qRT-PCR. Figure S7 shows PER1, PER2, CLOCK and BMAL1 expression in normal donors (ND, n = 24) in comparison to CLL samples from prognostic subgroups defined by CD38 expression (CD38+samples, n = 24 vs. CD38− samples, n = 27). mRNA expression is relative to GAPDH. Data are presented in a box-and-whisker format: the difference of the 25th and 75th percentile form the box (interquartile range, IQR), with the median marked as a line; the whiskers go down to the smallest value and up to the largest values. The Mann-Whitney U-test was used to compute p-values for pairwise comparisons. (TIF) [file pone.0034347.s007.tif]

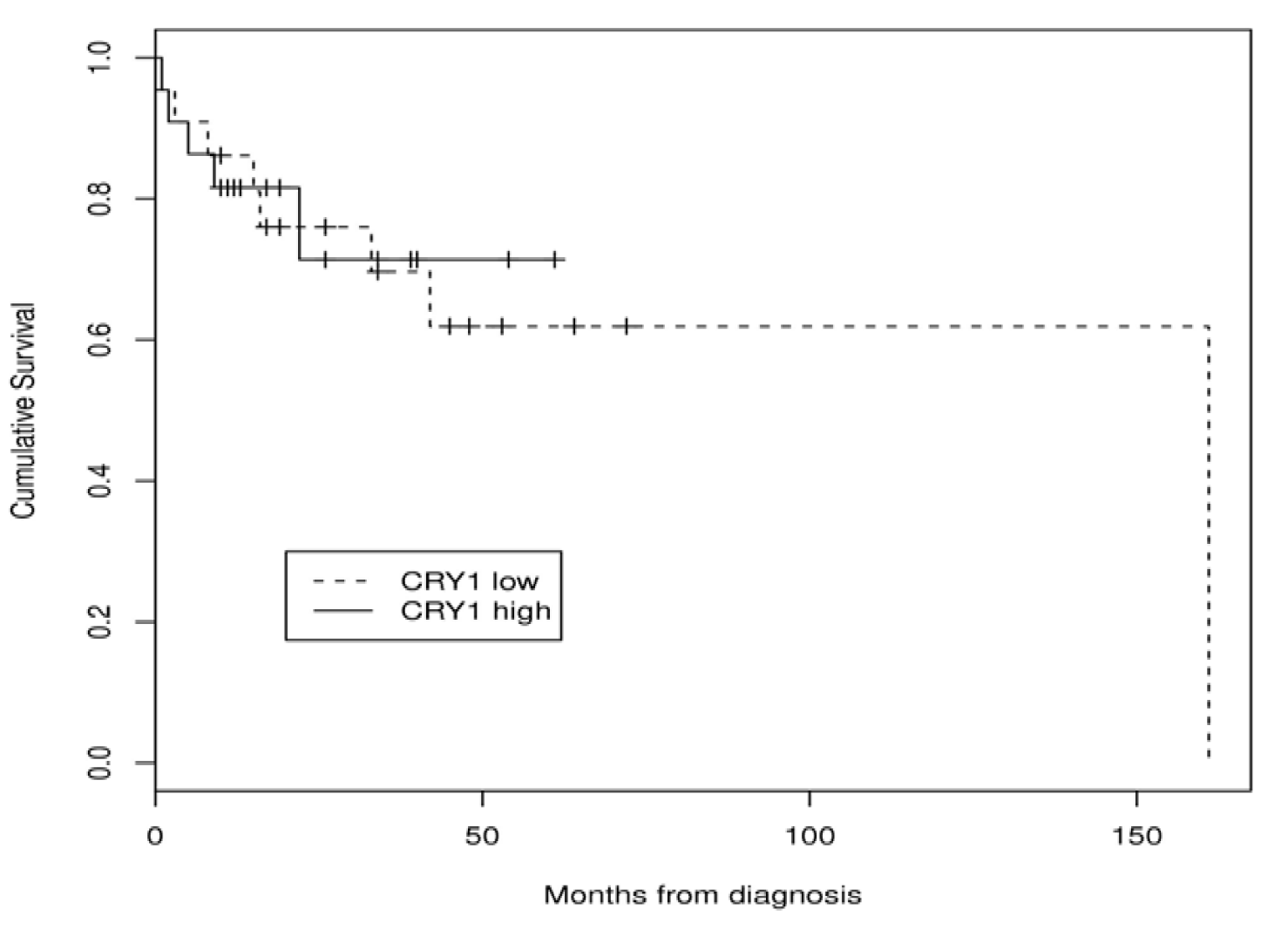

Supplement: Figure S8 — Kaplan-Meier curves for overall survival in ALL according to high and low expression of CRY1. The median CRY1 ΔCT value was used as a cut-off to define patient subgroups with high and low CRY1 mRNA transcript levels. Figure S8 illustrates the overall survival for 48 patients with ALL according to high and low expression of CRY1 mRNA levels. Statistical differences were analyzed by log-rank test. (TIF) [file pone.0034347.s008.tif]

**Table S3** Individual methylation profile (MassArray assay) and CRY1 expression values


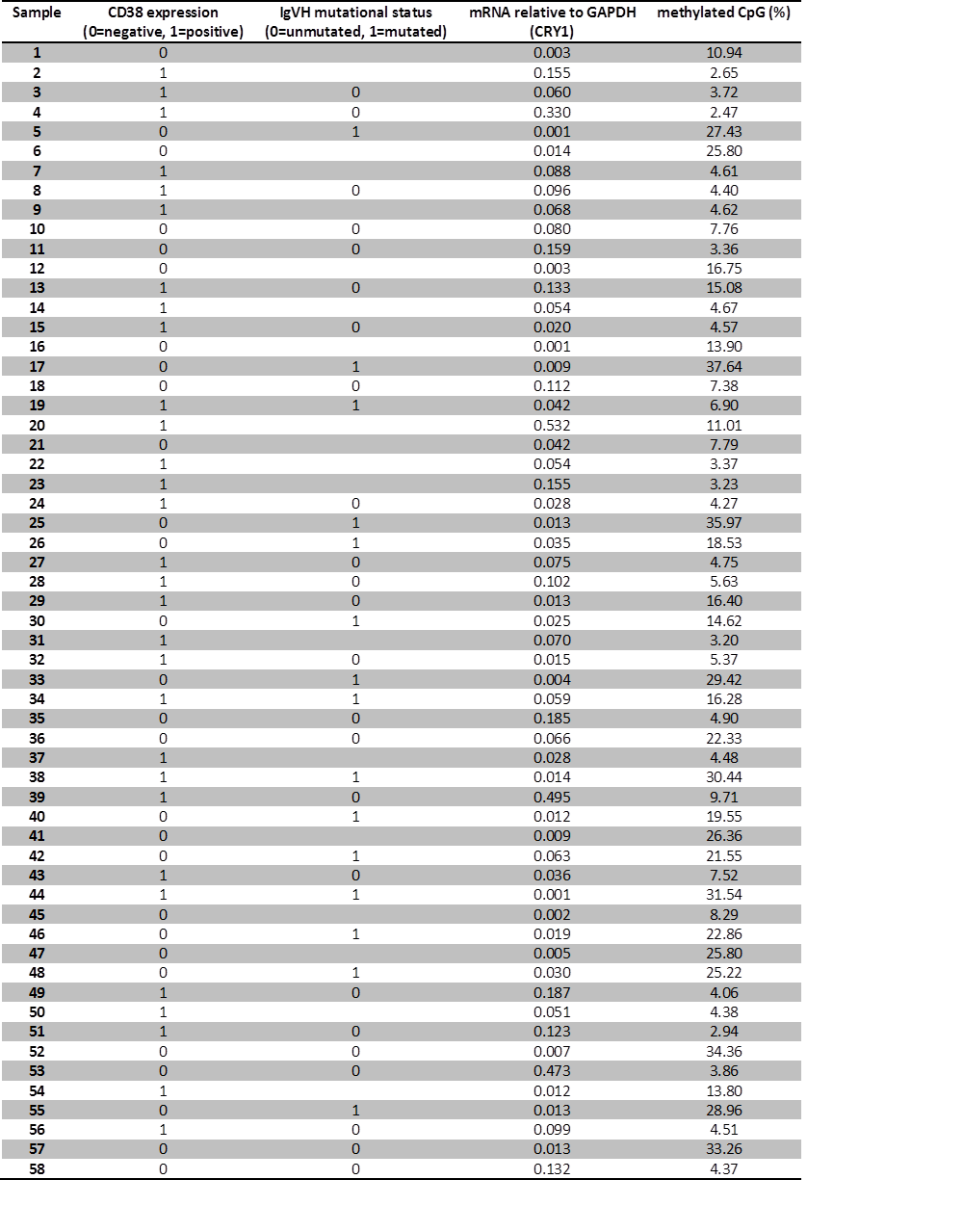

Supplement: Table S3 — Individual methylation profile (MassArray assay) and CRY1 expression values. (DOCX) [file pone.0034347.s011.docx]
