# Supplementary material for: Epigenetic Silencing of the Circadian Clock Gene CRY1 is Associated with an Indolent Clinical Course in Chronic Lymphocytic Leukemia
Source: PLoS One. 2012 Mar 28;7(3):e34347. doi: 10.1371/journal.pone.0034347 (PMC3314606; doi:10.1371/journal.pone.0034347)
Supplement: Table S1 — Characteristics of CLL patients tested for CRY1 promoter hypermethylation. (DOCX) [file pone.0034347.s009.docx]

**Table S1** Characteristics of CLL patients tested for CRY1 promoter hypermethylation

| **Parameter** |  | **No. of patients (%)** |
| --- | --- | --- |
| Total No. |  | 57 |
| Sex | male | 39 (68) |
|  | female | 18 (32) |
| Age, years | median | 62 |
|  | range | 36-86 |
| Follow-up, months | median | 68 |
| Binet Stage at diagnosis | A | 40 (70) |
|  | B | 11 (19) |
|  | C | 4 (7) |
|  | n.a. * | 2 (4) |
| Binet Stage at last follow-up | A | 12 (21) |
|  | B | 11 (19) |
|  | C | 9 (16) |
|  | n.a. * | 25 (44) |
| CD38 expression | < 20% | 27 (47) |
|  | ≥ 20% | 30 (53) |
| FISH cytogenetics | favorable | 34 (60) |
|  | unfavorable | 14 (25) |
|  | n.a. | 9 (15) |
| Treatment history | untreated | 31 (54) |
|  | treated | 24 (42) |
| IgVH mutational status | unmutated | 15 (26) |
|  | mutated | 13 (23) |
|  | n.a. * | 29 (51) |

* n.a., not available
