# Supplementary material for: Epigenetic Silencing of the Circadian Clock Gene CRY1 is Associated with an Indolent Clinical Course in Chronic Lymphocytic Leukemia
Source: PLoS One. 2012 Mar 28;7(3):e34347. doi: 10.1371/journal.pone.0034347 (PMC3314606; doi:10.1371/journal.pone.0034347)
Supplement: Table S2 — Clinical characteristics of ALL patients. (DOCX) [file pone.0034347.s010.docx]

**Table S2** Clinical characteristics of ALL patients

| **Parameter** |  | **No. of patients (%)** |
| --- | --- | --- |
| Total No. |  | 48(100) |
| Sex | male | 26 (54) |
|  | female | 22 (46) |
| Age, years | median | 40,4 |
|  | range | 17-80 |
| white blood cell (WBC) at diagnosis, x 10^9^/l | median | 107.51  (range 3.09-355) |
| Immunophenotypic Subtype |  |  |
| common ALL |  | 19 (40) |
| Pre-B ALL |  | 2 (4) |
| Pro-B ALL |  | 5 (10) |
| Mature B-ALL |  | 1 (2) |
| Burkitt type ALL |  | 3 (6) |
| early T-ALL |  | 5 (10) |
| mature T-ALL |  | 1 (2) |
| cortical T-ALL |  | 7 (15) |
| T-LBL |  | 3 (6) |
| T-ALL unclassified |  | 2 (4) |
| Follow-up (months) |  | 29 |
